# How national laws enhance palliative care integration: lessons from the Philippines, South Korea, and Taiwan

**DOI:** 10.7189/jogh.16.04230

**Published:** 2026-07-10

**Authors:** Laura Monzón-Llamas, Timo Carpen, Ednin Hamzah, Yinwei Wang, Rumalie Corvera, Agnes B Bausa-Claudio, Sujeong Kim, Carlos Centeno

**Affiliations:** 1 University of Navarra, ATLANTES Global Observatory of Palliative Care, Pamplona, Spain; 2 Helsinki University Hospital and University of Helsinki, Palliative Care Center, Helsinki, Finland; 3 University Health Network and University of Toronto, Department of Supportive Care, Toronto, Canada; 4 Hospis Malaysia, Kuala Lumpur, Malaysia; 5 Hualien Tzu Chi Hospital, Centre for Palliative Care, Hualien, Taiwan; 6 Tzu Chi University, Department of Medical Humanities, Hualien, Taiwan; 7 Asian Hospital and Medical Center, Supportive and Integrative Services, Manila, Philippines; 8 The Ruth Foundation for Palliative and Hospice Care, Philippines; 9 National Hospice and Palliative Care Council of the Philippines, Philippines; 10 Jose B. Lingad Memorial General Hospital, Palliative and Supportive Care Unit, San Fernando Pampanga, Philippines; 11 The Catholic University of Korea, Research Institute for Hospice/Palliative Care, Seoul, South Korea

**Keywords:** palliative care, legislation, law, development, system integration

## Abstract

**Background:**

Palliative care is increasingly recognised as a public health priority, yet its integration into national health systems remains uneven. Legal frameworks can enable or constrain access to services. This study examined how national laws influence palliative care integration in the Asia-Pacific region through a comparative analysis of legal frameworks in Taiwan, South Korea, and the Philippines.

**Methods:**

This two-phase multimethod study combined a regional assessment of palliative care development with legal document analysis. In Phase 1, country profiles were developed using the World Health Organization palliative care indicators framework. In Phase 2, national legislation was collected and analysed using a structured coding framework aligned with an expanded palliative care development model. Findings were validated through consultation with national experts.

**Results:**

Taiwan’s legislation provides a comprehensive and enforceable framework for palliative care, anchored in universal health coverage, and supported by strong governance and patient autonomy provisions. South Korea’s legislation prioritises end-of-life decision-making and institutional procedures but lacks community-based service mandates. The Philippines has advanced palliative care through broader health laws and insurance reforms, but the absence of a standalone law and weak enforcement continue to limit integration. Across all three countries, legal clarity, institutional support, education, and community engagement influenced the effectiveness of legal provisions.

**Conclusions:**

Legal frameworks can facilitate palliative care integration when they are binding, well enforced, and aligned with governance structures, health system capacity, and sociocultural context. Limited legal scope or weak implementation mechanisms are associated with fragmented access. Context-sensitive legislation is a key structural enabler of equitable palliative care within universal health coverage.

In recent decades, palliative care (PC) has gained global recognition as a vital component of modern health systems, offering comprehensive support for individuals with serious and life-limiting illnesses [1]. According to the World Health Organization (WHO), palliative care extends beyond the management of physical symptoms to encompass psychological, social, and spiritual support, with the goal of enhancing the quality of life for both patients and their families. Notably, evidence also suggests that palliative care can reduce the use of other healthcare resources – such as emergency care and hospitalisations in secondary healthcare – highlighting its potential not only to enhance well-being but also to provide cost-effective interventions that improve health system performance [2,3]. Despite its importance, access to palliative care remains uneven, particularly in the Asia Pacific region, where 70% of specialist PC services are concentrated in fewer than 15% of countries [4], leading the WHO to call for its integration into all levels of healthcare as a core element of universal health coverage [5].

Legislation and regulatory frameworks can play a crucial role in enabling such integration. The recognition of palliative care as a human right and an issue of health equity has further fuelled the development of diverse legal and regulatory approaches – ranging from general health laws to dedicated palliative care legislation and strategic national policies [6]. However, the enforceability and effectiveness of these laws vary considerably. Evidence indicates that specific laws with enforceable regulatory mechanisms tend to have the most significant impact in advancing palliative care development [7]. Palliative care laws act as both catalysts and structural foundations for the development of services and can shape the implementation of palliative care in diverse ways – either by guiding the transition from centralised, specialised services to accessible community-based models, or by scaling up community-led efforts into formalised national systems [8–10].

In this context, comparative legal analysis offers a structured method for examining how different countries use legal frameworks to advance palliative care integration. This study explores a shared legal question: how do national laws shape the delivery and institutionalisation of palliative care within diverse health systems? By analysing the legal frameworks of the Philippines, South Korea, and Taiwan, each representing distinct legislative traditions and health governance models, we aim to identify common elements, contextual barriers, and differences in how legislation affects the provision and accessibility of palliative care services.

## METHODS

To assess these legal frameworks systematically, the study employs the WHO’s ‘palliative care development house model’, a public health-based framework that outlines essential structural components – including policy, service provision, essential medicines, education and training, research, and community involvement. This model enables a multidimensional evaluation of how each country’s legal and regulatory environment supports national palliative care strategies and responds to the increasing global demand for quality care for individuals with serious and life-limiting illnesses [11].

**Figure 1:**
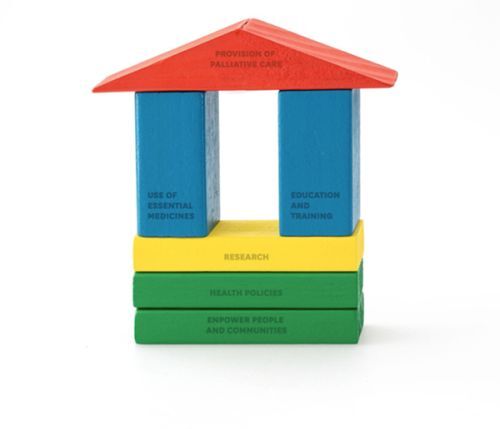
Conceptual framework for palliative care development based on the World Health Organization palliative care development house model.

The WHO palliative care development house model was used as the conceptual framework for analysis (**Figure 1**).

**Table 1** maps the selected indicators to the WHO conceptual framework.

**Table 1:** Relationship between the selected indicators and the World Health Organization conceptual framework for palliative care development

Dimension	#	Indicators for monitoring palliative care development
Empowerment of people and communities	1	Existence of groups dedicated to promoting the rights of patients in need of palliative care, their families, their caregivers and disease survivors
	2	Existence of national policy or guideline addressing advance care planning of medical decisions for the use of life-sustaining treatment or end-of-life care
Health policies	3	Existence of a current national palliative care plan, programme, policy or strategy with a defined implementation framework
	4	Inclusion of palliative care in the list of health services provided at the primary care level in the national health system
	5	Existence of a national coordinating authority for palliative care (labelled as unit, branch, department) in the Ministry of Health (or equivalent) responsible for palliative care
Research	6	Existence of congresses or scientific meetings at the national level specifically related to palliative care
	7	Palliative care research in the country estimated by peer-reviewed articles
Essential medicines	8	Reported annual opioid consumption, excluding methadone, in Defined Daily Dose for statistical purposes (S-DDD)
	9	Availability of essential medicines for pain and palliative care at all levels of care
	10	General availability of immediate-release oral morphine (liquid or tablet) at the primary level
Education and training	11	Proportion of medical and nursing schools with palliative care formal education in undergraduate curricula
	12	Specialisation in palliative medicine for physicians
Integrated palliative care services	13	Number of specialised palliative care services in the country per population
	14	Number of specialised paediatric palliative care services for children population in the country

A comparative systematic analysis was performed to examine national palliative care laws and their role for palliative care integration into primary health systems in the Philippines, South Korea, and Taiwan. The research was conducted in three phases between January 2023 and April 2025, using both qualitative and quantitative methods to ensure comprehensive and context-sensitive insights.

### Phase 1: regional assessment using WHO indicators

The first phase involved a regional evaluation of palliative care development using the WHO’s actionable indicators framework [12], which reflects a public health approach across six domains: national policies, service availability, access to essential medicines, education and training, community empowerment, and research. Each country developed and validated a national palliative care profile for the recently launched APHN Atlas of Palliative Care in the Asia Pacific Region 2025, which represents the most comprehensive regional data set currently available [4]. This cross-sectional, observational study integrated existing data with expert input, combining quantitative indicators with qualitative insights to enhance contextual relevance.

### Phase 2: legal mapping and document analysis

The second phase (2024–2025) focused on the identification, extraction, and comparative analysis of national palliative care laws in the Philippines, South Korea, and Taiwan. Full legislative texts, legislative summaries, and government reports were collected through official government websites, parliamentary databases: Philippines (Senate Legislative Information System), South Korea (Korean Law Information Center), and Taiwan (Ministry of Justice Law Database). Legal texts were verified by national experts directly involved in their drafting or implementation.

The document search used a combination of keywords (‘law’, ‘act’, ‘enactment’, ‘palliative care’, ‘hospice’, ‘life-sustaining treatment’, and ‘end-of-life’), in English and national languages, applying inclusion and exclusion criteria consistent with the framework described by Woitha *et al.* (2015). The legal analysis was complemented and contextualised by the country-level findings from Phase 1, which provided insight into how broader policy environments, workforce development efforts, access to opioids, and community engagement, either enhanced or constrained the integration of palliative care into national health systems.

A clear working definition of ‘national palliative care law’ guided inclusion, focusing on legislation enacted by national authorities that established rights and duties to ensure equitable access to palliative care. Eligible laws addressed service delivery, integration into health systems, or patient rights. Regional laws, general health laws without explicit PC content, and non-binding national strategies were excluded unless legally mandated.

### Analytical framework and data extraction

An expanded version of the WHO ‘palliative care development house model’ structured the analysis, covering six core dimensions – policy, medicines, education, service delivery, research, and community empowerment – supplemented by legal categories drawn from prior literature and expert input [9,10,13]. A structured coding framework was applied to assess 31 indicators across seven domains: country context, formal legal characteristics, substantive provisions, right to palliative care, alignment with WHO dimensions, related legislation, and mechanisms for implementation or follow-up.

A structured framework with 31 indicators across seven domains was used to guide legal analysis (**Table 2**).

**Table 2:** Structured framework for comparative analysis of national palliative care legislation: 31 indicators across seven domains

Domains	Indicators	Working definition
1. Country context	1. WHO Region	According to WHO Regional Offices
	2. Income classification	Income level
	3. Level PC development	Tripodoro et al. 2025
2. Formal legal characteristics	4. Year of enactment	Enacted by national authority or parliament.
	5. Law title	Original
	6. Law title	English
	7. Word count	Total number of words in the final version of the law.
	8. Structure	How the law is organised, major sections, chapters, and articles.
	9. Contents	Main topics regulated by the law
3. Substantive provisions	10. Purpose	Fundamental reason or motivation behind the law
	11. Objectives	Measurable goals
	12. Principles	Ethical, legal, and conceptual foundations
4. Right to PC	13. PC right	Right to receive palliative care (explicitly or implied)
	14. PC definition	Including textual quotes where applicable.
5. Alignment with WHO dimensions	15. Society	Promotion of participation of individuals, families, and communities in palliative care development and decision-making about their health.
	16. Governance	Leadership and policy framework for implementing and regulating palliative care, ensuring proper access and funding.
	17. Research	Research in palliative care to improve scientific evidence and care.
	18. PC medicines	Availability and access to palliative care essential medications
	19. Undergraduate education	Palliative care training into undergraduate educational programmes.
	20. Continuing education	Continuing education in palliative care
	21. Specialisation	Specialisation in palliative care for physicians and other professionals.
	22. Integration of services	Capacity of the national health systems to integrate primary and specialist palliative care services.
	23. Specialised services	Organisation and provision of specialised palliative care services for more complex patients.
6. Related legislation	24. Palliative sedation	Palliative sedation
	25. Advance Care Planning	Advance care planning and decision-making for patients
	26. Adaptation of therapeutic effort	Adaptation of medical and palliative interventions according to patient needs, preferences, and desires.
	27. Requests for assisted death	Euthanasia, assisted suicide, or hastening death.
7. Mechanisms for implementation	28. Standards	Norms and standards established by the law to ensure quality and consistency in palliative care.
	29. Financing	Funding mechanisms to cover the costs associated with implementing the law.
	30. Additional aspects	Any other relevant topics
	31. Subsequent regulations	Official documents issued after the original law to complement or develop its provisions.

PC – palliative care, WHO – World Health Organization

Legal texts were examined through comparative thematic analysis, with key provisions extracted and categorised. Direct quotations were used where needed to ensure precision and preserve legal meaning. Coding and thematic synthesis were performed manually using Excel and Word. Artificial intelligence ChatGPT (OpenAI) was used to assist in identifying specific quotations from national legal documents, for each indicator. All AI-assisted outputs were reviewed and validated by two independent researchers for contextual accuracy and adherence to the framework; all outputs were manually verified. AI was not used for data interpretation or to generate original scientific content.

### Expert validation

To strengthen validity, national experts reviewed the findings and provided context on each law’s development, objectives, implementation challenges, and real-world impact on health system integration. Experts were selected from the ATLANTES network based on their direct participation in palliative care law development or recognised national expertise. Each expert received an invitation letter, informed consent form, and semi-structured interview guide. Interviews were conducted individually (20–40 minutes) and analysed thematically. Ethical approval for expert validation interviews was obtained from the University of Navarra Research Ethics Committee.

Research team roles were defined as follows:

(a) Legal document retrieval – LM-LL, TC, CC;

(b) Coding and analysis – LM-LL, TC, CC;

(c) Expert validation – RC, AB-C, SK, and YW.

This integrated approach enabled a multidimensional comparison of national PC laws and provided actionable insights into how legal frameworks support or hinder the integration of palliative care into health systems across diverse Asia-Pacific contexts.

Reporting followed relevant EQUATOR guidance for observational and qualitative components. Checklist S1 is provided in the **Online Supplementary Document**.

## RESULTS

This comparative section presents the main legislative features and implementation trajectories of national palliative care laws in the Philippines, South Korea, and Taiwan. Each country illustrates a distinct stage of legal maturity and integration within health systems. The Philippines’ Hospice and Palliative Care Act (Senate Bill) [14] prioritises integration within primary care. South Korea’s Act on Hospice and Palliative Care and Decisions on Life-Sustaining Treatment for Patients at the End of Life [15] incorporates both care provision and end-of-life decision-making. Taiwan’s Hospice Palliative Care Act [16], one of the earliest such laws in Asia, represents a comprehensive and evolving legal model. Together, these cases offer critical insights into how legislation can support the delivery of community-based palliative care and improve system-wide accessibility.

### The Philippines' Hospice and Palliative Care Act

The Philippines' Hospice and Palliative Care Act was drafted in 2015 and passed by the lower house in 2016, but it has not yet been approved by the Senate, preventing its enactment into law. Despite repeated efforts – most recently in 2022 – multiple versions of the bill have stalled at various stages of deliberation [14,17]. Leaders from national PC associations continue to work closely with the Department of Health (DOH) to advocate for the law’s passage, seeing it as essential for unlocking sustainable funding and institutional support. While the standalone PC law remains pending, PC provisions have been successfully embedded in the Universal Health Care Act and the National Integrated Cancer Control Act, both enacted in 2019 [18,19]. These laws, along with the 2016 DOH Administrative Order that established the National Policy on Palliative and Hospice Care, form the current regulatory backbone for palliative care in the country [20].

The proposed law adopts an inclusive definition of palliative care, extending its scope beyond terminal illnesses to include patients with chronic, complex, or debilitating conditions, thereby promoting early and proactive palliative care integration. Its core objectives focus on improving quality of life, alleviating suffering, and prioritising symptom management and psychosocial support.

The legislation mandates both national government and healthcare institutions to establish and maintain hospice and palliative care services. Hospitals are required to develop referral networks with continuity of care, while local government units (LGUs) must implement home-based PC programmes, embedding community-based services into the broader health system. Although community empowerment is not a major legislative focus, national and local advocacy groups actively support patient rights and the promotion of palliative care initiatives.

In terms of workforce development, the law calls for the inclusion of palliative care in medical and nursing school curricula and mandates continuing education for various healthcare professionals and volunteer workers [14]. Despite this provision, implementation remains inconsistent: only three out of 70 medical schools currently require palliative care as a core subject, while most offer it as an elective. Similar trends are observed in nursing education. Efforts by the Commission on Higher Education (CHED) are under way to integrate palliative care more fully, yet specialised services and expertise remain limited [21].

A critical gap lies in access to essential palliative care medicines. The law does not specifically address the availability of opioids, and restrictive regulations by the Philippine Drug Enforcement Agency [22], a shortage of licensed prescribers, and excessive bureaucracy continue to hinder pain management – especially in rural areas. Furthermore, primary care facilities often lack secure systems for handling-controlled medications. These barriers contribute to unequal access and underutilisation of necessary treatments.

Although the proposed legislation outlines a comprehensive, system-wide approach to palliative care, its lack of legal enactment results in limited implementation and sustainability. Despite this, notable progress has been made through the release of the Manual of Operations, Procedures and Standards for National Palliative and Hospice Care Program (2021), training module development, and the finalisation of clinical practice guidelines in 2023 [23]. However, without a formal legal mandate to ensure accountability, resource allocation, and enforcement – particularly regarding opioid availability – these efforts remain fragile. Regulatory and logistical barriers continue to constrain equitable access to pain relief, and service delivery relies heavily on under-resourced community-led initiatives.

In contrast to the Philippines’ pending legislation and fragmented policy implementation, South Korea’s law provides a clear national framework, albeit with narrow clinical reach and a primarily procedural orientation.

### South Korea’s Act on Hospice and Palliative Care

South Korea’s Act on Hospice and Palliative Care and Decisions on Life-Sustaining Treatment for Patients at the End of Life, enacted in 2016, was largely shaped by two landmark cases. The 1997 ‘Boramae Incident’, where a physician was criminally charged for withdrawing life-sustaining treatment at a family's request, created significant legal uncertainty and deterred clinicians from providing palliative care. Public demand for dignified end-of-life options intensified after the 2009 ‘Grandma Kim’ case, in which the Supreme Court, for the first time, authorised the removal of ventilator support from a patient in a persistent vegetative state, formally arguing the right to die with dignity. These two events collectively catalysed strong advocacy within the medical and legal communities for protective legislation [24,25]. In response, the 2016 law prioritised legal clarity over palliative system development, establishing protocols for advance directives and physician orders for life-sustaining treatment, and aiming to shield providers from prosecution [26].

The law frames hospice care as the central focus and defines palliative care narrowly limiting access to patients with cancer, Acquired Immunodeficiency Syndrome, Chronic Obstructive Pulmonary Disease, or liver cirrhosis. Despite several amendments, the law remains focused on procedures for patients in imminent dying phase, overlooking essential palliative care provisions such as pain and symptom management for those who are not yet at that stage. According to national experts, this legislative approach has inadvertently stalled efforts to broaden access to earlier-stage palliative care and community-based services.

The legislation establishes a centralised care model through National and Regional Hospice Centers, supporting care delivery in hospices, hospitals, and at home. Financial coverage under the National Health Insurance ensures service accessibility. Although the 2nd National Plan for Hospice and Life-sustaining Treatment (2024–2028) aims to enhance hospice services, it does not promote palliative care integration at the primary care level [27]. The law also mandates structured training for healthcare professionals and supports the development of dedicated hospice institutions. While undergraduate education is not addressed directly, since 2016, palliative care has been a required component of medical education in all 40 medical schools, albeit with variation in content and depth.

While the law underscores symptom control, it lacks detailed provisions on opioid access. Nevertheless, under the Narcotics Control Act, authorised healthcare professionals can prescribe opioids [28]. Access to pain medication is high in urban areas, with over 90% of primary care facilities stocked.

Although the act mandates the establishment of National and Regional Hospice Centres and guarantees financial coverage through National Health Insurance, its hospital-centric structure remains a barrier to community integration. Pilot programmes under the Community Integrated Care Act aim to bridge this gap but are still in early stages [29].

While South Korea’s legislation emphasises terminal care and legal protection, Taiwan’s legal framework embeds palliative care principles into the broader health insurance and long-term care systems.

### Taiwan’s Hospice Palliative Care Act

Taiwan’s Hospice Palliative Care Act, first enacted in 2000 and revised in 2013 and 2021, is centred on patient autonomy, granting terminally ill individuals the legal right to refuse life-sustaining treatment [16]. Originally conceived as a ‘Natural Death Act’ by pioneers’ advocates, the law was renamed to reflect Taiwanese cultural sensitivities around death. It was complemented by the Patient Right to Autonomy Act (2016), although the overlap between these laws has occasionally created ambiguity [30]. Importantly, the Cancer Control Act (2003) – which mandates that at least 50% of deceased cancer patients should have received palliative care – was cited by national experts as a more influential catalyst for expanding palliative care access [31].

Taiwan’s Hospice Palliative Care Act defines hospice palliative care as supportive care aimed at relieving pain and suffering at the end of life. While the law does not primarily aim to expand service access, it ensures the legal enforceability of patient decisions, making Taiwan’s approach uniquely person-centred among the three countries studied.

While the law does not prescribe direct strategies for systemic integration, Taiwan has successfully embedded palliative care within Universal Health Coverage (UHC) through the National Health Insurance (NHI) system. Services are available at all levels of care and integrated into the National Long-Term Care 2.0 initiative [32]. A strong consultative model has evolved, especially in hospitals, to ensure that palliative care is offered even where full specialist services are not feasible. Interviewees emphasised that legal reform alone would not have achieved this level of integration without parallel movements in advocacy, insurance reimbursement, academic development, and faith-based community engagement.

The law also underpins a national advance care planning framework. Patients’ preferences are digitally stored in their NHI cards, making them accessible through electronic health records. This system facilitates informed, person-centred decisions at the point of care [33]. While palliative care education is not legally mandated, most medical schools include it in their core curricula, helping to normalise end-of-life conversations among younger generations.

Access to palliative care medicines is not explicitly addressed in the law but is supported through NHI reimbursement policies. Primary care physicians can prescribe essential medications, including opioids, and access is enhanced by rural health stations and widespread availability of oral morphine at district and regional hospitals. Service provision is overseen by multiple agencies within the Ministry of Health and Welfare, including regulatory, accrediting, and promotional bodies [34].

Stakeholders stressed that the success of Taiwan’s system stems from the simultaneous development of legal frameworks, training, advocacy, and a robust payment structure – each reinforcing the other to ensure that palliative care is not only available but also ethically, culturally, and financially sustainable.

Comparatively, these three legislative trajectories reveal how distinct political, cultural, and economic contexts shape the scope and effectiveness of national palliative care laws. The Philippines demonstrates how the absence of enactment limits accountability and financing; South Korea shows how narrowly focused legal protection can constrain system-wide integration; and Taiwan exemplifies how coherent legal alignment with financing and education mechanisms supports sustainable palliative care development. This comparative synthesis underscores that legislative maturity alone is insufficient – effective integration depends on legal enforceability, institutional capacity, and financial continuity.

A detailed comparative summary of legal characteristics across countries is provided in Table S1 in the **Online Supplementary Document**.

## DISCUSSION

This comparative analysis of national palliative care laws in the Philippines, South Korea, and Taiwan reveals a spectrum of legislative maturity, policy integration, and implementation effectiveness in advancing PC within health systems. Across all three countries, the study highlights how the presence or absence of a dedicated legal framework – and the extent to which it is operationalised through implementation mechanisms – shapes the institutionalisation and equity of access to palliative care services.

Differences in palliative care access across the three countries cannot be explained by legislation alone but rather by the interaction between legal frameworks, health financing systems, and political structures. Taiwan’s integrated National Health Insurance provides stable funding and institutional support, enabling palliative care to become a routine part of oncology and hospital services. In South Korea, although universal coverage exists, the Life-Sustaining Treatment Decision Act (2016) arose from legal-ethical debates rather than system-wide planning, resulting in a hospital-based, procedurally oriented model with limited integration into community or primary settings. In the Philippines, the absence of a dedicated palliative care law leaves financing fragmented and dependent on shifting administrative priorities.

Collectively, these comparisons show how differing governance approaches shape the pathways through which palliative care becomes institutionalised. The interplay between financing mechanisms, political stability, and cultural acceptance thus determines the extent to which legislation translates into tangible, equitable access to palliative care services.

These findings also carry broader implications for global health policy, particularly in low- and middle-income countries seeking to integrate essential services into Universal Health Coverage (UHC). Legal frameworks reinforced by coordinated health financing, advocacy efforts and dedicated governance structures, as seen in Taiwan, can facilitate the system-wide integration of palliative care, while tools like the WHO’s actionable indicators offer practical benchmarks for tracking progress toward SDG Target 3.8 on equitable service access and system integration.

In the Philippines, the lack of formal enactment of the Hospice and Palliative Care Act has hindered sustainable national investment and implementation, despite the inclusion of provisions within broader laws such as the Universal Health Care Act and the National Integrated Cancer Control Act. The proposed law defines PC inclusively and envisions multi-level service integration, but its impact remains limited by legislative delays and inconsistent operationalisation. This fragmentation undermines workforce development, limits service decentralisation, and exacerbates opioid access barriers. Politically, the absence of binding legislation with clear enforcement mechanisms, leaves programmes exposed to disruption due to changes in government leadership or administrative priorities, while culturally, ongoing advocacy by civil society actors has helped sustain momentum despite policy inertia.

South Korea presents a contrasting model, where the 2016 Act on Hospice and Palliative Care and Decisions on Life-Sustaining Treatment has institutionalised legal protections for terminally ill patients and healthcare providers, particularly through mechanisms like advance directives and physician orders for life-sustaining treatment. However, the law narrowly defines eligibility for palliative care services and places significant emphasis on formal documentation processes – such as registering advance decisions and completing legal forms – rather than promoting broader, system-wide integration of services. As a result, palliative care remains highly centralised, with limited expansion into primary or community-based care. Although training is required and services are widely available in urban areas, access faces more challenges in rural areas. Culturally, hesitancy to appear permissive of euthanasia has contributed to restrictive legal eligibility criteria, driven by ethical concerns about potential overlap between euthanasia and terms such as ‘dying process’ and ‘end-of-life process’, as well as prior debates on withdrawal of life-sustaining treatment. Politically, the system’s efficiency is supported by a centralised governance model, but flexibility for community integration is limited.

Taiwan demonstrates effective palliative care integration through a coherent legal framework anchored by the Hospice Palliative Care Act and Patient Right to Autonomy Act, supported by its inclusion in the National Health Insurance system. This foundation is strengthened by structured advance care planning, digital health records, and coordinated oversight by government and health agencies. The data underscore how legal structure shapes both access and quality in palliative care – countries with enforceable and well-aligned laws, like Taiwan, demonstrate higher levels of service integration and institutional accountability [35], while those with absent or narrowly scoped laws, such as in the Philippines or South Korea, continue to face systemic challenges despite policy intent.

In this sense, the Taiwanese legal target that at least 50% of people dying from cancer should receive palliative care stands out as uniquely concrete among the palliative care legislation identified in our global study – none of the laws we identified establish such a specific coverage threshold – and can be regarded as a highly relevant minimum benchmark for integration, since population-based studies confirm that reaching and even surpassing this level is feasible in systems with adequately organised palliative care services [36].

Legal texts across all three countries lack explicit provisions on opioid regulation, but this gap has the most significant impact in the Philippines, where regulatory and logistical barriers continue to limit access to essential pain medications. Addressing this issue should be a key focus of future reforms, alongside broader efforts to strengthen legal frameworks. The Philippines and South Korea could benefit from incorporating elements of Taiwan’s model – such as decentralised service delivery, integration of palliative care into primary care, and mandated advance care planning within legal and digital systems [37]. Reforms should also expand legal definitions beyond narrow disease-based eligibility and adopt culturally sensitive approaches to foster public support and legislative acceptance.

Overall, this comparative synthesis shows that legislative effectiveness depends on alignment between legal frameworks, financing mechanisms, and institutional accountability. It underlines the value of analysing palliative care legislation not only by content but also by its functional interaction with the broader health system.

Ultimately, palliative care laws are not merely symbolic instruments; they function as practical tools for integrating compassionate, person-centred care into national health systems. Legislative design, enforcement, and alignment with broader health governance mechanisms critically determine whether palliative care remains a marginalised service or evolves into a fundamental component of health equity and universal health coverage in the Asia-Pacific region. Across contexts, common challenges include insufficient legal provisions for palliative care medication – particularly opioid access – limited support for community-based care, and variable enforcement mechanisms. Nonetheless, each country demonstrates that incremental legal advancements – when aligned with health system realities and cultural values – can lay the foundation for broader PC integration. Policymakers should prioritise legally binding strategies that ensure education, funding, accountability, and decentralisation.

## CONCLUSIONS

This comparative analysis highlights how legal frameworks shape the integration, equity, and accessibility of palliative care in diverse health systems. Taiwan’s model demonstrates how enforceable legislation, combined with universal coverage, robust governance, and multi-sectoral collaboration, supports comprehensive integration. South Korea offers strong protections for end-of-life decisions but lacks breadth in community-based care. The Philippines, while incorporating palliative care into broader health laws, continues to face challenges due to the absence of a fully enacted national law. These cases collectively underscore that binding, well-implemented legislation – aligned with cultural, political, and health system contexts – is essential for embedding palliative care as a core component of compassionate and equitable healthcare systems.

**Data availability:** All data supporting the findings of this study are included in the manuscript and its accompanying tables and figure. The legal and policy sources analysed are publicly available from the official governmental and parliamentary websites of the Philippines, South Korea, and Taiwan, as cited in the text. Additional research tools can be obtained from the corresponding author on reasonable request and with the agreement of the contributing country experts.

## Additional material


Online Supplementary Document
