# Supplementary material for: How national laws enhance palliative care integration: lessons from the Philippines, South Korea, and Taiwan
Source: J Glob Health. 2026 Jul 10;16:04230. doi: 10.7189/jogh.16.04230 (PMC13351595; doi:10.7189/jogh.16.04230)
Supplement: Online Supplementary Document [file jogh-16-04230-s001.pdf]

Supplement to: Monzón-Llamas L, Carpen T, Hamzah E, Wang Y, Corvera R, Bausa-Claudio A, Kim S, Centeno C. How national laws enhance palliative care integration: lessons from the Philippines, South Korea, and Taiwan. J Glob Health. 2026;16:04230.

Checklist S1. Reporting guidance for the observational and qualitative components of the study

Title: How National Laws Enhance Palliative Care Integration: Lessons from the Philippines, South Korea, and Taiwan

Reporting followed relevant EQUATOR guidance for the observational and qualitative components of this mixed-methods study. The observational component was mapped to relevant STROBE items, while the expert validation component was mapped to selected COREQ items.

This study used a mixed-methods design combining:

- a cross-sectional regional assessment of palliative care development using WHO actionable indicators (Phase 1);
- comparative legal document analysis of national palliative care laws (Phase 2);
- expert validation through semi-structured interviews.

This checklist maps the relevant reporting items to the corresponding sections of the manuscript.

STROBE items for the observational components

STROBE item	Recommendation	Where addressed in manuscript
1. Title and abstract	Indicate study design and provide balanced summary	Title; Abstract (Methods and Results)
2. Background/rationale	Explain scientific background and rationale	Introduction, paragraphs 1–3
3. Objectives	State specific objectives	Introduction, final paragraph
4. Study design	Present key design elements early	Methods, first paragraph
5. Setting	Describe setting, locations, and relevant dates	Methods, overall study design (January 2023–April 2025; Philippines, South Korea, Taiwan)
6. Participants / study units	Describe country selection and eligibility	Methods, Phase 1 and Phase 2

7. Variables / domains	Define outcomes, legal domains, and indicators	Methods, WHO indicators and analytical framework
8. Data sources / measurement	Describe sources and collection methods	Methods, Phase 1 regional assessment; Phase 2 document search
9. Bias	Describe efforts to address bias and improve validity	Methods, expert validation; AI-assisted outputs independently reviewed
10. Study size	Explain scope and rationale	Methods, three-country comparative design; APHN Atlas dataset
11. Quantitative variables	Explain handling of indicators	Methods, Phase 1 WHO actionable indicators
12. Analytical methods	Describe legal and thematic analysis methods	Methods, analytical framework and data extraction
13. Participants / study units	Report numbers and units included	Methods and Results, three countries; national experts involved
14. Descriptive data	Present contextual characteristics	Results, country-specific sections
15. Outcome data	Report main findings	Results
16. Main results	Summarize comparative findings	Results, comparative synthesis
17. Other analyses	Report subgroup / additional analyses	Not applicable
18. Key results	Summarize key findings	Discussion, opening paragraph
19. Limitations	Discuss limitations and potential bias	Discussion
20. Interpretation	Provide overall interpretation	Discussion and Conclusions

21. Generalisability	Discuss transferability / external validity	Discussion
22. Funding	State funding and role of funders	Funding section

Selected COREQ items for the expert validation interviews

**COREQ item**	**Recommendation**	**Where addressed in manuscript**
Research team and reflexivity	Identify researchers involved and roles	Methods, Research team roles
Participant selection	Describe sampling strategy and rationale	Methods, Expert Validation
Setting	Describe interview context and participants	Methods, national experts from ATLANTES network
Data collection	Describe interview mode and duration	Methods, interviews conducted individually (20–40 min)
Interview guide	Mention prompts / guide used	Methods, invitation letter, consent form, and semi-structured interview guide
Data analysis	Describe thematic review and validation process	Methods, expert validation and thematic analysis
Participant checking	Describe review / verification of findings	Methods, national experts reviewed findings

Some checklist items were interpreted proportionally to reflect the multimethod design of this study, which combined cross-sectional assessment, comparative legal document analysis, and expert validation interviews. Items not applicable to this design were not reported.

Table S1. Comparative analysis of national palliative care laws in South Korea, the Philippines, and Taiwan across 31 indicators

Indicators	*Working Definition*	Philippines	South Korea	Taiwan
**1. WHO Region**	*According to WHO Regional Offices*	WPRO	WPRO	WPRO
**2. Income classification**	*Income level*	LMIC	HIC	HIC
**3. Level pc development**	*Tripodoro et al. 2025*	GDS 2143: Progressing	GDS 3036: Established	GDS 3846: Advanced
**4. Year of enactment**	*Enacted by national authority or parliament.*	Drafted in 2015, passed by Lower House in 2016, not yet enacted by Senate.	2016 (amended in 2018 and 2020, 2021, 2023, 2025).	2000 (amended in 2021)
**5. Law title**	*Original*	"An Act Integrating Hospice and Palliative Care into the Philippine Health Care System".	"호스피스 및 완화의료와 말기환자에 대한 연명치료 결정에 관한 법률"	“安寧緩和醫療條例”
**6. Law title**	*English*	"An Act Integrating Hospice and Palliative Care into the Philippine Health Care System".	"Act on Hospice and Palliative Care and Decisions on Life-Sustaining Treatment for Patients at the End of Life"	“Hospice Palliative Care Act”
**7. Word count**	*Total number of words in the final version of the law.*	1306	8243	1186
**8. Structure**	*How the law is organized, major sections, chapters, and articles.*	18 sections addressing policy, accreditation, quality assurance, services, training, research, funding, and regulations.	Six chapters: General Provisions, Management and Implementation of Life-Sustaining Treatment, Hospice and Palliative Care, Supplementary Provisions, Penalties, and addenda for enforcement and transitions.	15 articles structured into sections addressing general provisions, patient rights, healthcare responsibilities, non-application of LST, penalties, and final provisions.
**9. Contents**	*Main topics regulated by the law*	Palliative care services, accreditation, training, expanded PhilHealth benefits, and research funding.	Palliative care, life-sustaining treatment, and related ethical oversight	Patients’ will on the medical treatment and patient´s right. Palliative care to alleviate suffering
**10. Purpose**	*Fundamental reason or motivation behind the law*	Ensure access to quality hospice and palliative care for patients with life-threatening illnesses, focusing on improving life quality and alleviating suffering (Section 2).	Protect the dignity and value of human beings by ensuring the best interests of patients at the end of life and respecting their self-determination. It addresses the need for comprehensive care for terminal patients, including the provision of hospice and palliative care, (Article 1).	Respect the autonomy of terminally ill patients by ensuring they have the right to make informed decisions about their medical treatment.
**11. Objectives**	*Measurable goals*	Ensure universal hospice and palliative care services in hospitals.<br>Establish accreditation and quality standards<br>Expand PhilHealth coverage for palliative care.<br>Promote education and training for healthcare professionals.<br>Support research and funding for indigent-serving institutions	Improve hospice care.<br>Expand services and train professionals.<br>Support families and ensure ethical oversight.<br>Promote public awareness through Hospice Day (Articles 6, 14, 21).	Ensure patient autonomy by providing a framework for making informed treatment decisions.<br>Improve quality of life through appropriate palliative care.<br>Ensure healthcare providers respect patients documented treatment wishes.

		(Sections 6, 4, 11, 8, 9, 12).		
**12. Principles**	*Ethical, legal, and conceptual foundations*	Principles of patient rights, holistic care, equity, and professionalism in providing quality palliative services (Sections 2, 3, 4, and 6).	Principles of dignity, autonomy, and best interests of patients (Article 1, 3). Informed decision-making and ethical oversight through ethics committees (Article 3, 14).	Principles of patient autonomy and the right to make informed decisions regarding treatment. Obligation of healthcare professionals to respect these decisions.
**13. PC right**	*Right to receive palliative care (explicitly or implied)*	*"The State guarantees the right of the people to quality health care and ensures that the health of the people is protected over the entire life cycle."*	It implicitly recognizes the right to receive palliative care by ensuring that terminal and end-of-life patients receive comprehensive hospice care, including pain relief and symptom management (Article 2, Item 6; Article 3).	It emphasizes to respect terminal illness patients' will on the medical treatment and protect their right.
**14. PC definition**	*Including textual quotes where applicable.*	As an "approach that improves the quality of life of patients with life-threatening, complex, and chronic illnesses or those experiencing progressively debilitating diseases beyond any benefit from curative treatment". It involves prevention and relief of suffering through early identification, assessment, and management of pain and symptoms (Section 3).	As medical care for terminal patients, focusing on physical, psychosocial, and spiritual treatment, including pain and symptom relief (Article 2, Item 6).	As "mitigatory and supportive medical care given to relieve terminal illness patients from physical, mental and spiritual pain, to improve their quality of life."
**15. Society**	*Promotion of participation of individuals, families, and communities in palliative care development and decision-making about their health.*	Not explicit mention of participation in decision making.<br>It mentions that immediate family members or relatives assigned<br>to provide hospice and palliative care to a relative<br>shall be allowed to use all existing leave benefits	It promotes participation by involving patients and families in decision-making about palliative care (Article 3, 17). Not any mention to community involvement.<br>but it encourages awareness of palliative care through initiatives like Hospice Day (Article 6).	It encourages participation of individuals and families in decision making process by allowing patients to document their treatment preferences (Article 4) and involving family members in decision-making when patients are unable to express their wishes (Article 8). Families can also act as medical surrogate agents, ensuring their role in palliative care decisions (Article 5).
**16. Governance**	*Leadership and policy framework for implementing and regulating palliative care, ensuring proper access and funding.*	The Department of Health, in partnership with the National Hospice and Palliative Care Council of the Philippines, leads the implementation and regulation of palliative care, ensuring accreditation, quality, and sustainability through the Office for Technical Services (Sections 4, 5, and 10).	The Minister of Health and Welfare (Article 7) and the National Hospice and Palliative Care Committee (Article 8), oversee the implementation, regulation, and funding of palliative care. The National Agency for Management of Life-Sustaining Treatment ensures proper management of care (Article 9).	Leadership and policy framework for implementing and regulating palliative care through the central competent authority, which is the Department of Health of the Executive Yuan.
**17. Research**	*Research in palliative care to improve scientific evidence and care.*	Provisions for research are focused on the Department of Health working with the Philippine Council for Health Research and Development to ensure ongoing research and data collection on hospice and palliative care, as well as securing	Provisions for developing research projects on hospice care (Article 21, Item 3) and fostering institutions specialized in hospice care (Article 25). It also mandates the collection and analysis of data related to terminal patients and hospice services (Article 23).	Not mentioned in the law text.

		funds for these activities (Section 9).		
**18. Medicines**	*Availability and access to palliative care essential medications*	Not mentioned in the law text.	It does not explicitly mention specific medications, but it ensures that pain management and symptom control are integral to hospice care (Article 2, Item 6). It also encourages the development of guidelines for symptom management.	Not mentioned in the law text.
**19. Undergraduate education**	*Palliative care training into undergraduate educational programs.*	It mentions that the Commission on Higher Education ensure the inclusion of courses on the principles and practice of hospice and palliative care in the curricula for medicine, nursing, and allied health courses (Section 8).	Not mentioned in the law text.	Not mentioned in the law text.
**20. Continuing education**	*Continuing education in palliative care*	It mandates ongoing training and continuing education for healthcare professionals, through programs developed by the Department of Health and the National Hospice and Palliative Care Council of the Philippines (Section 8).	It includes provisions for continuing education in palliative care (Article 21, Item 4).	Not mentioned in the law text.
**21. Specialization**	*Specialization in palliative care for physicians and other professionals.*	Law mandates the organization of continuing training programs for health professionals, health workers and volunteers in palliative care. However, specialization is not mentioned.	It promotes specialization in palliative care by fostering institutions specialized in hospice care and training professionals in the field (Article 21, Item 4).	Not mentioned in the law text.
**22. Integration of services**	*The national health system's capacity to meet the needs of individuals experiencing health-related suffering, integrating primary and specialized care services.*	It mentions that Hospitals must link to a referral and aftercare network organized by local governments under the DOH's guidance, and rural health units must develop home-based palliative care programs with local hospices (Section 6).	Not mentioned in the law text.	Not mentioned in the law text.
**23. Specialized services**	*Organization and provision of specialized palliative care services for more complex patients.*	Not mentioned in the law text.	It supports the development of specialized care centres (Article 25).	Not mentioned in the law text.
**24. Palliative sedation**	*Palliative sedation*	Not mentioned in the law text.	Not mentioned in the law text.	Not mentioned in the law text.
**25. Advance care planning**	*Advance care planning and decision-making for patients*	Not mentioned in the law text.	It supports advance care planning through life-sustaining treatment plans and advance statements (Articles 10, 11, 12), allowing decisions to be	It allows patients to document their treatment preferences (Article 4). If patients are unable to express their wishes, a medical surrogate or family

			made in advance or by family if the patient is incapacitated (Article 17).	member can make decisions on their behalf (Articles 5 and 8). These decisions are recorded in the national health insurance card (Article 6).
**26. Adaptation of therapeutic effort**	*Adaptation of medical and palliative interventions according to patient needs, preferences, and desires.*	Not mentioned in the law text.	It ensures patients can express their wishes regarding life-sustaining treatments and hospice care through life-sustaining treatment plans and advance statements (Articles 10, 11, 12). Healthcare professionals must respect and adjust interventions according to the patient's informed decisions (Article 3).	It ensures that medical and palliative interventions are adapted to patient preferences by documenting their choices (Article 4). If the patient cannot express their wishes, family members or surrogates can make decisions based on prior preferences (Articles 5 and 8).
**27. Requests for assisted death**	*Euthanasia, assisted suicide, or hastening death.*	Not mentioned in the law text.	Not mentioned in the law text.	Not mentioned in the law text.
**28. Standards**	*Norms and standards established by the law to ensure quality and consistency in palliative care.*	Required accreditation (Section 4), quality assurance (Section 5), and mandatory palliative care services in hospitals and rural health units (Section 6).	Guidelines for symptom management (Article 21). Ethics committees for oversight (Article 14). Evaluation of hospice institutions (Article 29). Comprehensive plans for care and training (Article 7).	Requirement for healthcare providers to inform patients and families about treatment options (Article 9) and document decisions in medical records (Article 9). Procedures for withholding life-sustaining treatment based on patients' wishes or family consent (Articles 7-8).
**29. Financing**	*Funding mechanisms to cover the costs associated with implementin g the law.*	Expanded PhilHealth benefits (Section 11). Financial assistance from the PCSO for nonprofit hospice institutions (Section 12). Tax exemptions on donations (Section 13). Appropriations from the DOH's budget (Section 14).	The Minister of Health and Welfare may fully or partially subsidize expenses incurred in hospice services to institutions specialized in hospice care. Financing of Hospice and palliative care is listed in another law.	Not mentioned in the law text.
**30. Additional aspects**	*Any other relevant topics*	It includes provisions for the creation of rules and regulations for implementation (Section 15).	It addresses additional topics such as, data protection for sensitive health information (Article 31, 32), and penalties for non-compliance with regulations (Articles 39–43).	The law includes penalties for non-compliance by healthcare providers (Articles 9-11).
**31. Subsequent regulations**	*Official documents issued after the original law to complement or develop its provisions.*	N/A.	Act No. 15542 (2018), Act No. 15912 (2018), and Act No. 17218 (2020), along with Presidential Decrees, complement and refine the original law's provisions on palliative care and life-sustaining treatment. Enforcement Decree of the National Insurance Act, where financing of Hospice and palliative care is included in Article 21.	The Hospice Palliative Care Act was amended on January 20, 2021.
